# Altered proteomics profile in the amnion of patients with oligohydramnios

**DOI:** 10.14814/phy2.14381

**Published:** 2020-02-28

**Authors:** Cecilia Y. Cheung, Robert A. Brace

**Affiliations:** ^1^ Department of Obstetrics and Gynecology Center for Developmental Health Oregon Health and Sciences University Portland OR USA

**Keywords:** amniotic fluid volume, intramembranous transport, metabolism, proteomics, transport signaling, vesicular trafficking

## Abstract

In pregnancy, idiopathic oligohydramnios is an obstetrical complication that compromises maternal health with poor perinatal outcome. Effective therapeutic treatment of this condition has been hampered by the unknown etiology and lack of understanding of cellular and molecular mechanisms that underlie idiopathic oligohydramnios. Amniotic fluid volume (AFV) is determined by intramembranous (IM) transport of amniotic fluid across the amnion and this pathway is regulated to maintain AFV within the normal range. To gain understanding of the causes of idiopathic oligohydramnios, we performed proteomics analysis of the human amnion to investigate the changes in protein expression profiles of cellular transport pathways and regulators in patients with oligohydramnios. Placental amnions from five patients with normal pregnancies and five patients with oligohydramnios were subjected to proteomics experiments followed by bioinformatics analysis. Using Ingenuity Pathway Analysis (IPA) software, five categories of biological functions and multiple canonical pathways within each category were revealed. The top differentially expressed proteins that participate in mediating these pathways were identified. The functional pathways activated include: (a) cellular assembly and organization, (b) cell signaling and energy metabolism, and (c) immunological, infectious, and inflammatory functions. Furthermore, the analysis identified the category of pathways that facilitate molecular endocytosis and vesicular uptake. Under oligohydramniotic conditions, the mediators of clathrin vesicle‐mediated uptake and transport as well as intracellular trafficking mediators were up‐regulated. These findings suggest that idiopathic oligohydramnios may be associated with alternations in cellular organization and immunological functions as well as increases in activity of vesicular transport pathways across the amnion.

## Graphical abstract

The proteomics profile in placental amnion of normal and oligohydramniotic patients revealed changes in activity of several categories of cellular pathways and modulators associated with low amniotic fluid volume. Alterations in expression of the key regulators of vesicular endocytosis and intracellular trafficking pathways may affect amniotic fluid transport across the amnion leading to amniotic fluid volume disorders including oligohydramnios.

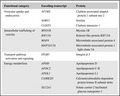

## 1 INTRODUCTION

Normal amniotic fluid volume (AFV) is necessary for proper growth and development of the fetus. Abnormalities in AFV occur in 7%–10% of ~4 million births in the US annually. An excess or deficiency in the volume of this fluid can cause obstetrical problems that compromise maternal and fetal well‐being. A majority (>70%) of these cases is diagnosed as oligohydramnios (reduced amniotic fluid volume). Conditions of fetal renal agenesis (Spiro et al., 2015), obstructive uropathy, preterm premature rupture of fetal membranes (PPROM), gestational hypertension and postdates are known causes of oligohydramnios. However, approximately 50% of the cases are of unknown origin. Oligohydramnios is associated with poor development of the fetal lung, pulmonary hypoplasia, premature delivery, IUGR (intrauterine growth restriction), umbilical cord compression, and increased risk of cesarean section (Locatelli et al., 2004). Development of oligohydramnios is more prevalent during the third trimester of pregnancy and clinical management of low AFV generally involves ultrasound monitoring and increased maternal fluid intake. However, treatment modalities are generally temporary with minimal long‐term efficacy largely due to the lack of understanding of the mechanisms that cause the reductions in AFV. The objective of the present study was to address this knowledge gap.

Experimental studies in animal models led to the current concept that amniotic fluid is a dynamic reservoir maintained within the normal range through regulation of inflow and outflow pathways (Brace, Anderson, & Cheung, 2014, 2018). At late gestation, fetal urine is the primary source of amniotic fluid while fetal swallowing removes fluid from the amniotic compartment (Brace et al., 2018). The intramembranous (IM) pathway allows transfer of fluid and dissolved solutes through the placental amnion from the amniotic cavity into the underlying fetal blood vessels on the surface of the placenta. This is a unidirectional pathway in that the majority of fluid flow is outward from the amniotic compartment while inward movement is negligible (Brace, Anderson, et al., 2014; Cheung & Brace, 2008). Previous research indicated that this IM pathway is the primary determinant of AFV (Brace et al., 2018). Furthermore, the placental amnion rather than the chorion determines the rate of IM transport and, as such, is the rate‐limiting layer and site of AFV regulation (Adams, Choi, Cheung, & Brace, 2005). Recent evidence suggests that IM transport rate is regulated by stimulators derived from fetal urine (Anderson, Jonker, Louey, Cheung, & Brace, 2013) and inhibitors from fetal membranes (Brace, Cheung, & Anderson, 2014). However, the identity of these regulators is currently not known. Intramembranous transport of amniotic fluid has been observed in late‐gestation fetal rhesus monkeys (Gilbert, Eby‐Wilkens, & Tarantal, 1997), and has been investigated and found to occur in humans (Mann, Nijland, & Ross, 1996).

Few studies have explored the possible causes of idiopathic oligohydramnios. In patients with oligohydramnios, amniotic expressions of the transmembrane water channel proteins aquaporin (AQP) 1 and AQP3 were found to be reduced (Zhu et al., 2009). In animal studies, the effect of fetal urinary prostaglandin E_2_ on IM transport was explored but results did not support such a role (Cheung, Beardall, Anderson, & Brace, 2014). Conversely, AQP1 appeared to play a positive role in regulating the rate of IM transport (Cheung, Anderson, & Brace, 2016) while the growth and permeability factor vascular endothelial growth factor (VEGF) may mediate amniotic fluid transport across the amnion (Cheung, Anderson, & Brace, 2017). Recently, we reported that IM transport across the amnion is a multifactorial regulatory process that involves cellular transport pathways and signaling modulators. Alterations in individual components could potentially affect the rate of transport. Furthermore, the mediators of intracellular trafficking appear to play an important role in determining the rate of transport across the IM pathway (Cheung, Anderson, & Brace, 2019). The transport process is characterized as vesicular transcytosis and is regulated by multiple uptake and intracellular trafficking pathways that are energy dependent (Sharshiner, Brace, & Cheung, 2017).

Previous studies have reported proteomics analysis of the human amnion for the identification of clinically significant biomarkers of problematic pregnancies (Hopkinson, McIntosh, Shanmuganathan, Tighe, & Dua, 2006; Park et al., 2006). A recent study utilized proteome analysis of the human placenta and amniotic fluid to explore the protein expression pattern in patients with polyhydramnios (Cen et al., 2020). However, there has been no study on proteomics analysis of the human amnion in conditions of oligohydramnios. The aim of the present study was to gain insights into the possible causes of idiopathic oligohydramnios. The design of the study was to investigate the protein expression profiles of IM transport regulators in the amnion of subjects with normal pregnancy at late gestation. In addition, we examined whether changes in expression of these transport mediators occurred in conditions of oligohydramnios. We tested the hypothesis that reduced AFV is associated with up‐regulation of vesicular transport and intracellular trafficking regulators. This would lead to enhanced IM transport rate and increased fluid removal from the amniotic compartment, resulting in AFV reduction. Large‐scale proteomics analysis methodology was used to determine protein expression profiles in the human amnion. Placental amnion but not reflected amnion was used for the study because transport of amniotic fluid and solutes through the IM pathway occurs across the placental amnion while minimally across the reflected amnion (Brace, 1995).

## 2 MATERIALS AND METHODS

### 2.1 Study population

The study design was approved by the Institutional Review Board of Oregon Health and Science University (OHSU). Written Informed Consent and HIPPA Research Authorization was obtained from all patients prior to participation in the study. Five patients with normal pregnancies and five patients with oligohydramnios were recruited at Labor and Delivery at OHSU. Oligohydramnios was defined by ultrasound assessment as an amniotic fluid index (AFI) of <8 cm and a single deepest pocket of <2 cm (Phelan, Smith, Broussard, & Small, 1987). Inclusion criteria were: singleton gestation, near‐term pregnancy, delivery via cesarean section, absence of active labor, and maternal age ≥18 years. Subjects with chorioamnionitis, fetal anomalies, or other pregnancy complications were excluded from the study. An AFI was obtained by the attending physician on the day of the cesarean section or within 2 weeks prior to delivery. Gestational age at delivery ranged from 37 to 39 weeks.

### 2.2 Amnion tissue collection

The amnion was obtained aseptically immediately upon delivery of the placenta at the time of cesarean section. The placental amnion was isolated by separation from the underlying chorionic plate and trimmed circumferentially 1 cm from the edge of the placenta and medially at least 1 cm from the umbilical cord insertion. Placental amnion but not reflected amnion was collected for study. Immediately after collection, the amnion was rinsed in sterile DMEM/F12 tissue culture medium (Invitrogen, Life Technologies) to remove blood and other contaminants. The amnion samples were snap‐frozen in liquid nitrogen and stored at −80°C until analysis. The samples were de‐identified and codified after collection.

### 2.3 Proteomics analysis by tandem mass tag methodology

Amnion tissues were extracted for total protein and submitted to OHSU Proteomics Shared Resource core facility for quantitative proteomics studies using tandem mass tag (TMT) technology. Sample processing and analysis have been described in our recent publication (Cheung et al., 2019). Following protein quantification by the bicinchoninic acid protein assay (BCA assay, Thermo Fisher Scientific), the samples were trypsin digested and TMT labeled for liquid chromatography–mass spectrometry‐mass spectrometry (LC/MS/MS, Dionex UltiMate 3000 UHPLC and Orbitrap Fusion Tribrid Mass Spectrometer, Thermo Fisher Scientific). Pooled amnion protein samples from normal (*n* = 5) and oligohydramniotic (*n* = 5) patients were included in the analysis as internal reference and analyzed in parallel with study samples. Normalization between TMT experiments were performed using the internal reference scaling (IRS) method with the pooled internal reference channels (Plubell et al., 2017). Peptide fragments were sequenced for protein identification. The peptide sequence data were processed using Proteome Discoverer software (version 1.4, Thermo Fisher Scientific) with SEQUEST and Percolator. Unique peptide identifications with Percolator q‐scores less than 0.05 and delta masses less than 20 PPM were used to sum reporter ions into protein totals. Differential protein abundance between groups was determined by comparing the IRS‐normalized total reporter ion intensities between experimental groups using the Bioconductor package edgeR. Comparisons between groups were performed using Fisher's exact test.

### 2.4 Bioinformatics and statistical analysis

The values in the normal and oligohydramnios groups were compared using unpaired *t*‐test and expressed as mean ± *SE*.

The proteomics datasets were subjected to robust bioinformatics analysis by the OHSU/ONPRC (Oregon National Primate Research Center) Bioinformatics & Biostatistics core service. The protein data was referenced to the Genome Reference Consortium for Homo sapiens, release 38 (GRCh38, 20,444 sequences) with Ensembl annotations. Normalized counts for all samples were merged into one file and imported into Bioconductor's (v3.1) DESeq2 to determine differential expression. Because DESeq2 ignored low count proteins when calculating the multiple‐comparison‐adjusted *p*‐value, no filtering of low count molecules was performed prior to the analysis. The only factor included in the statistical model was experimental condition. For comparisons between the normal and oligohydramniotic conditions, expression fold change was determined as the ratio of condition 2/condition 1, where condition 2 was oligohydramnios and condition 1 was normal AFV. Log2 fold changes and *p* values were adjusted for false discovery rate (FDR) using the procedure of Benjamini and Hochberg (1995). In the results, a statistical *p* value of <.1 was used as the significance cut‐off for the comparisons.

For biological function assignment and pathway analysis, the entire proteomics dataset was applied to Ingenuity Pathway Core Analysis (IPA, version 27821452, June 2016, Qiagen Bioinformatics). The analysis was based on the Ingenuity Knowledge Base, a thoroughly curated data repository on biological interactions and functional annotation derived from relevant and extensive knowledge in the literature and multiple bioinformatics databases. Using the IPA’s Pathway Analysis module for functional annotation and analysis, biological functions were identified by category. Relevant canonical pathways were selected and Z‐scores were determined for individual canonical pathways as a predictor of pathway activation. The datasets were then subjected to analysis by the Multiple Group Comparison module of IPA to determine differential expression of proteins between the normal and oligohydramniotic groups.

## 3 RESULTS

In this study, the groups of normal pregnant patients and those with oligohydramnios were maternal age‐matched and gestational age‐matched. The mean gestational age of the normal group was 38.7 ± 0.4 weeks (*n* = 5) and was not different from the 38.1 ± 0.6 weeks (*n* = 5) in the oligohydramniotic group (Table 1). The AFI in the normal group was 13.0 ± 1.0 cm, within the acceptable range of 8–25 cm for normal near‐term pregnancy (Phelan et al., 1987). In the oligohydramniotic group, AFI averaged 4.5 ± 0.5, significantly lower than that in the normal group (*t* = 7.50, *p* < .0001) and within the clinically defined criteria of <8 cm for oligohydramnios.

**Table 1:** Gestational age (GA) and amniotic fluid index (AFI) of study subjects

Normal		Oligohydramnios	
GA (week/day)	AFI	GA (Week/day)	AFI
37.0	15.6	38.6	4.8
39.2	10.6	38.4	5.5
39.1	13.6	38.0	5.5
39.3	14.6	36.1	3.3
39.1	10.6	39.4	3.6

### 3.1 Proteomics expression in human placental amnion

Proteomics experiments identified a total of 3,476 unique proteins expressed in the human placental amnion of normal and oligohydramniotic patients. However, following bioinformatics and statistical analysis, the number of differentially expressed proteins was considerably reduced. A panel of 446 differentially expressed proteins (12.8% of total proteins) was obtained when comparisons were made between normal and oligohydramniotic conditions.

The entire proteomics datasets from normal and oligohydramniotic groups were uploaded onto IPA for statistical comparison and pathway analysis. The differentially expressed proteins were functionally annotated followed by categorization and pathway assignment. The top 10 up‐regulated and top 10 down‐regulated proteins were identified. Table 2 lists the top differentially expressed proteins with their respective encoding genes. The expression fold change of this panel of proteins ranged from increases of 1.52 fold to decreases of 0.62 fold.

**Table 2:** Ingenuity pathway analysis identified top differentially expressed proteins in placental amnion of patients with oligohydramnios

Protein	Encoding gene	Fold change*, *p* value
Top 10 up‐regulated		
Formin‐like protein 3	*FMNL3*	**↑** fold change = 1.524, .001
Pregnancy zone protein	*PZP*	**↑** fold change = 1.466, .002
Protein FAM49A	*FAM49A*	↑ fold change = 1.454, .001
Ig heavy constant gamma‐3	*IGHG3*	↑ fold change = 1.431, .001
Fibulin‐1	*FBLN1*	↑ fold change = 1.389, .001
Calponin‐3	*CNN3*	↑ fold change = 1.376, .006
Desmoglein‐3	*DSG3*	↑ fold change = 1.368, .011
Flavin reductase (NADPH)	*BLVRB*	↑ fold change = 1.357, .006
Sorting nexin‐21	*SNX21*	↑ fold change = 1.353, .004
Cytochrome C oxidase subunit 5B	*COX5B*	↑ fold change = 1.348, .016
Top 10 down‐regulated		
Aminopeptidase *N*	*ANPEP*	↓ fold change = 0.623, .001
ADAMTS‐like protein 4	*ADAMTSL4*	↓ fold change = 0.628, .001
Serpin B12	*SERPINB12*	↓ fold change = 0.631, .001
Zymogen granule protein 16 homolog B	*ZG16B*	↓ fold change = 0.649, .001
Frizzled‐1	*FZD1*	↓ fold change = 0.667, .001
Perilipin‐2	*PLIN2*	↓ fold change = 0.671, .001
Caspase 14	*CASP14*	↓ fold change = 0.673, .001
Proteasome assembly chaperone 4	*PSMG4*	↓ fold change = 0.684, .002
Plakophilin 2	*PKP2*	↓ fold change = 0.688, .001
Dermcidin	*DCD*	**↓** fold change = 0.695, .003

* The increase (**↑**) or decrease (**↓**) in expression fold change and *p* value for each protein. Log2 fold change data were converted to fold change values for presentation. Fold change was calculated as the ratio of oligohydramnios/normal values.

### 3.2 Pregnancy associated functional pathways in human placental amnion

Using the IPA Core Analysis module and overlaying with the Ingenuity Knowledge Base, pregnancy associated biological functions, physiological and disease states, relevant canonical pathways, and differentially expressed protein mediators were revealed. Five main functional categories emerged from the IPA analysis with each category consisting of multiple canonical pathways (Table 3): (a) Cell assembly and organization. This category of functions regulates actin‐mediated cytoskeletal organization and assembly, cell‐cell adhesion, and cell migration. The pathways involved are actin nucleation by ARP‐WASP complex, regulation of actin‐based motility by Rho, and Rac signaling. In the amnion of the oligohydramniotic patients, these pathways were activated as predicted by the positive Z‐scores. Among the top differential expressed proteins, eight proteins participate in these pathways with three proteins up‐regulated and four down‐regulated. (b) Cell signaling and energy metabolism. These cellular functions activate transcriptional regulation, signal transduction, and lipid and glucose metabolism. The pathways included in this category are integrin signaling, PI3K signaling, PI3K/AKT signaling, SAPK/JNK signaling, sphingosine‐1‐phosphase signaling, HIF1α signaling, PPARα/RXRα activation, and PTEN signaling. Five of these pathways were activated while the PTEN pathway was suppressed and two with no change in activity. Of the proteins that modulate these pathways, three were up‐regulated and four down‐regulated. (c) Immunological, infectious, and inflammatory functions. These pathways initiate immunological responses to viral infection of cells, and mediate inflammatory responses to foreign antigens. The assigned pathways are B cell receptor signaling, intrinsic prothrombin activation pathway, NF‐ ƙB activation, IL‐6 signaling, acute phase response signaling, and glucocorticoid receptor signaling. As predicted, these pathways were activated with the exception of acute phase response signaling, which was suppressed in activity, and glucocorticoid receptor signaling with no change in activity. Among the proteins participating in these pathways, three were up‐regulated while two were down‐regulated. (d) Molecular endocytosis and vesicular uptake. These pathways serve important functions in macromolecule uptake, receptor‐mediated endocytosis, and phagocytosis. Within this category, the selected pathways are macropinocytosis signaling, clathrin‐mediated endocytosis signaling, phagosome formation, and virus entry via endocytic pathways. For these pathways, there were no overall changes in activity as predicted by the zero Z‐score. The mediators of these functions include one protein that was up‐regulated and four were down‐regulated. (e) Developmental and hereditary disorders. These pathways are implicated in cell proliferation, hereditary malignancy, and cancer development. The two pathways involved are embryonic stem cell pluripotency and hereditary breast cancer signaling. The former pathway was activated while no change in activity was predicted for the latter pathway. Two of the proteins which modulate these functions were up‐regulated while two were down‐regulated.

**Table 3:** Categories of biological functions in placental amnion of normal and oligohydramniotic patients

Canonical pathway		Protein (Gene)	
Name	*Z*‐score*	Up‐regulated	Down‐regulated
Cellular assembly and organization			
Cellular function: cytoskeletal organization, cell‐cell adhesion, cell motility, cell migration			
Actin nucleation by ARP‐WASP complex	+	Formin‐like protein 3 (*FMNL3*)	Aminopeptidase *N* (*ANPEP*)
Regulation of actin‐based motility by Rho	+	Fibulin 1 (*FBLN1*)	ADAMTS‐like protein 4 (*ADAMTSL4*)
Rac signaling	+	Desmoglein 3 (*DSG3*)	Serpin B12 (*SERPINB12*)
			Plakophilin 2 (*PKP2*)
Cell signaling and energy metabolism			
Cellular function: transcriptional regulation, signal transduction, lipid and glucose metabolism			
Integrin signaling	+	Flavin reductase (*BLVRB*)	Zymogen granule protein 16 homolog B (*ZG16B*)
PI3K signaling	+	Sorting nexin 21 (*SNX21*)	Perilipin 2 (*PLIN2*)
PI3K/AKT signaling	+	Cytochrome C oxidase subunit 5B (*COX5B*)	Proteasome assembly chaperone 4 (*PSMG4*)
SAPK/JNK signaling	+		Plakophilin 2 (*PKP2*)
Sphingosine‐1‐phosphate signaling	+		
HIF1α signaling	0		
PPARα/RXRα activation	0		
PTEN signaling	−		
Immunological, infectious and inflammatory functions			
Cellular function: viral infection of cells, inflammation, inflammatory responses			
B cell receptor signaling	+	Pregnancy zone protein (*PZP*)	Caspase 14 (*CASP14*)
Intrinsic prothrombin activation pathway	+	Ig heavy constant gamma 3 (*IGHG3*)	Dermcidin (*DCD*)
NF‐ƙB activation	+	Calponin 3 (*CNN3*)	
IL‐6 signaling	+		
Acute phase response signaling	−		
Glucocorticoid receptor signaling	0		
Molecular endocytosis and vesicular uptake			
Cellular Function: Macromolecule uptake, receptor‐mediated endocytosis, phagocytosis			
Macropinocytosis signaling	0	Sorting nexin 21 (*SNX21*)	Aminopeptidase *N* (*ANPEP*)
Clathrin‐mediated endocytosis signaling	0		Zymogen granule protein 16 homolog B (*ZG16B*)
Phagosome formation	0		Perilipin 2 (*PLIN2*)
Virus entry via endocytic pathways	0		Dermcidin (*DCD*)
Developmental and hereditary disorders			
Cellular Function: Cell proliferation, hereditary malignancy, cancer development			
Embryonic stem cell pluripotency	+	Fibulin 1 (*FBLN1*)	Frizzled 1 (*FZD1*)
Hereditary cancer signaling	0	Calponin 3 (*CNN3*)	Caspase 14 (*CASP14*)

* Z‐score: +, positive Z‐score; −, negative Z‐score; 0, no detectable change in activity.

### 3.3 Transport associated differentially expressed proteins in placental amnion of oligohydramniotic patients

Further analysis of the proteomics datasets of normal and oligohydramniotic groups uncovered a panel of 13 differentially expressed proteins that are known mediators of vesicular transport, intracellular trafficking, transport signaling, and energy metabolism. Under conditions of oligohydramnios, nine proteins were up‐regulated and four were down‐regulated (Table 4). Expressions of this group of proteins ranged from increases of 1.320 fold to decreases of 0.798 fold. As shown in Table 5, this panel of differentially expressed proteins could be categorized into mediators for: (a) Vesicular uptake and endocytosis. These include clathrin‐associated adaptor protein 1, and clathrin interactor 1. (b) Intracellular trafficking of vesicles. The five proteins participating in this function are kinesin‐like protein KIF 13A, 2 microtubule‐associated proteins, sortilin, and myosin‐1B. (c) Transport pathway activation and signaling. The mediating protein identified is integrin β‐5. (d) Energy metabolism. The five mediators are solute carrier‐2 facilitated glucose transporter 1, calcium/calmodulin‐dependent protein kinase II subunit delta, and the 3 apolipoproteins.

**Table 4:** Differentially expressed transport associated proteins in placental amnion of oligohydramniotic patients

Protein	Encoding transcript	Fold change*, *p* value
Up‐regulated		
Clathrin‐associated adaptor protein 1 subunit mu‐2	*APIM2*	↑ fold change = 1.320, .001
Kinesin‐like protein KIF13A	*KIF13A*	↑ fold change = 1.280, .001
Apolipoprotein D	*APOD*	↑ fold change = 1.266, .033
Microtubule‐associated protein 1 light chain 3A	*MAP1LC3A*	↑ fold change = 1.235, .081
Apolipoprotein L1	*APOL1*	↑ fold change = 1.224, .100
Apolipoprotein C‐II	*APOC2*	↑ fold change = 1.223, .098
Microtubule‐associated protein 4	*MAP4*	↑ fold change = 1.214, .017
Sortilin	*SORT1*	↑ fold change = 1.189, .011
Clathrin interactor 1	*CLINT1*	↑ fold change = 1.104, .046
Down‐regulated		
Myosin‐1B	*MYO1B*	↓fold change = 0.798, .066
Integrin β‐5	*ITGB5*	↓ fold change = 0.816, .033
Solute carrier‐2 facilitated glucose transporter 1	*SLC2A1*	↓ fold change = 0.837, .036
Calcium/calmodulin‐dependent protein kinase II subunit delta	*CAMK2D*	↓ fold change = 0.857, .015

* The increase (↑) or decrease (↓) in expression fold change and *p* value for each protein. Log2 fold change data were converted to fold change values for presentation. Fold change was calculated as the ratio of oligohydramnios/normal values.

**Table 5:** Functional categories of differentially expressed proteins in placental amnion of oligohydramniotic patients.

Functional category	Encoding transcript	Protein
Vesicular uptake and endocytosis	*AP1M2*	Clathrin‐associated adaptor protein 1 subunit mu‐2
	*CLINT1*	Clathrin interactor 1
Intracellular trafficking of vesicles	*MYO1B*	Myosin‐1B
	*KIF13A*	Kinesin‐like protein KIF13A
	*MAP4*	Microtubule‐associated protein 4
	*MAP1LC3A*	Microtubule‐associated protein 1 light chain 3A
	*SORT1*	Sortilin
Transport pathway activation and signaling	*ITGB5*	Integrin β‐5
Energy metabolism	*APOD*	Apolipoprotein D
	*APOC2*	Apolipoprotein C‐II
	*APOL1*	Apolipoprotein L1
	*CAMK2D*	Calcium/calmodulin‐dependent protein kinase II subunit delta
	*SLC2A1*	Solute carrier‐2 facilitated glucose transporter 1

## 4 DISCUSSION

In order to better understand the causes of idiopathic oligohydramnios, the present study was launched to explore the changes in proteomics profile in the amnion under conditions of oligohydramnios. Our results indicated that 12.8% of the proteins identified in the amnion of oligohydramniotic patients were altered in expression. Based on IPA analysis, changes in expression of this panel of differentially expressed proteins are associated with modifications in biological functions and activities of cellular pathways. Among the 446 proteins that were differentially expressed, the top 10 up‐regulated proteins and top 10 down‐regulated proteins were selected by the IPA analysis. These proteins regulate cellular pathways including those involved in cell assembly and signaling, infection and inflammation, molecular transport and developmental disorders. Furthermore, the IPA analysis revealed three categories of biological functions that were activated under oligohydramniotic conditions. In the category of cell assembly and organization, three canonical pathways and seven proteins are found to be associated with these functions. The proteins formin‐like protein 3, fibulin 1, and desmoglein 3 were up‐regulated, while aminopeptidase N, ADAMTS‐like protein 4, serpin B12, and plakophilin 2 were down‐regulated. These proteins regulate actin polymerization and bundle assembly, cytoskeletal organization, cell‐cell adhesion, cell motility, and migration. For the category of cell signaling and energy metabolism, eight signal transduction pathways and seven proteins are involved. Among the proteins, flavin reductase, sorting nexin 21, and cytochrome C oxidase subunit 5B were up‐regulated, while zymogen granule protein 16 homolog B, perilipin 2, proteasome assembly chaperone 4, and plakophilin 2 were down‐regulated. This group of proteins initiates transcriptional regulation, signal transduction, receptor kinase activation as well as glucose and lipid metabolism. Within the category of immunological, infectious, and inflammatory functions, there were six pathways with five participating proteins. The proteins pregnancy zone protein, immunoglobulin heavy constant gamma 3, and calponin 3 were up‐regulated, and caspase 14 together with dermcidin were down‐regulated. These pathways and proteins mediate immunological responses to inflammation and infection from foreign antigens such as viral proteins. An additional category selected was developmental and hereditary disorders with one pathway activated and the other showing no change in activity. Of the four participating proteins, fibulin1 and calponin 3 were up‐regulated, while frizzled 1 and caspase 14 were down‐regulated. These are known mediators of embryonic stem cell potency and cell proliferation.

One category of biological function selected by IPA analysis was molecular endocytosis and vesicular uptake. The four pathways and five proteins involved mediate the process of endocytosis, the initial step in vesicular uptake and internalization of extracellular macromolecules including foreign antigens. However, the analysis predicted no change in activity for this function under oligohydramniotic conditions. Recently, based on transcriptomics and proteomics analysis of the ovine amnion, we reported that transcellular transport of amniotic fluid across the ovine amnion is mediated by multiple endocytotic and transcellular transport pathways (Cheung et al., 2019). The differentially expressed transcripts and proteins identified are categorized into four groups of mediators that participate in: (a) vesicular uptake and endocytosis, (b) intracellular trafficking of vesicles across the cell, (c) transport pathway activation and signaling, and (d) energy metabolism required for active transport. Importantly, the molecules that participate in intracellular trafficking are the key regulators of IM transport. In the present study, we further examined the proteomics datasets based on the findings in the ovine amnion and isolated a panel of 13 transport‐associated mediators that were differentially expressed in placental amnion of oligohydramniotic patients. These proteins modulate vesicular transport pathways and changes in expression of these proteins potentially could affect amniotic fluid transport. Similarly, this group of proteins can be organized into 4 categories. The group of intracellular trafficking mediators were kinesin‐like protein KIF 13A, microtubule‐associated protein 1 light chain 3A, microtubule‐associated protein 4, and sortilin. Kinesins are microtubule‐dependent motor proteins which mediate transcellular trafficking along microtubules (Shima et al., 2018), and microtubule‐associated proteins regulate assembly and turn‐over of microtubules which serve as cargo transport routes (Cabrales Fontela et al., 2017). Sortilin is involved in intracellular protein transport. The up‐regulation of these mediators suggests that intracellular trafficking could be activated under oligohydramniotic conditions. The category of vesicular endocytosis mediators includes two proteins that regulate the activities of clathrin‐coated vesicles. These are clathrin‐associated adapter protein 1 subunit mu‐2 and clathrin interactor 1. Adaptor protein complexes 1 and 2 (AP‐1 and AP‐2) are essential for recruitment of clathrin molecules for vesicle coat assembly, cargo selection, and vesicle transport (Park & Guo, 2014). Clathrin interactor 1 facilitates cargo transport via clathrin‐coated vesicles (Mills et al., 2003). The increased expression of these two molecules indicates that clathrin vesicle‐mediated transport may be augmented in oligohydramnios. In contrast, under the category of transport activation and signaling, the multifunctional signaling protein integrin β‐5 (Luo, Carman, & Springer, 2007) was down‐regulated. In the category of energy metabolism, the modulators including solute carrier‐2 facilitated glucose transporter 1 and calcium/calmodulin‐dependent protein kinase II subunit delta were down‐regulated, whereas the 3 apolipoproteins were up‐regulated. Collectively, the results suggest that transport and trafficking pathways as well as lipid metabolism were activated despite suppressed cell signaling and glucose transport activities. As such, these findings support our initial hypothesis that reduced AFV may be associated with up‐regulations of vesicular transport and intracellular trafficking regulators in oligohydramnios.

The IPA analysis provided new insights into the major biological functions within the amnion cell during pregnancy and the changes in activity of cellular pathways during conditions of oligohydramnios. In addition, the analysis identified the key regulators that were differentially expressed. Individual modulators presumably would participate in various components within each pathway. Although some of these molecules overlap in their functions, the observed phenotypic reduction in AFV apparently results from the net change in effect of the key regulators. Taken together, these analyses suggest that oligohydramnios is likely associated with increases in activity of cytoskeletal assembly and organization as well as cellular immune function. The up‐regulation of these functions likely would be mediated by activations of the signal transduction and transcription pathways.

The present study generated important understanding in the etiology of AFV disorders. However, there are inherent pitfalls within the study. The sample size of study subjects was limited thus reducing the statistical power of the data obtained. Another limitation of the study was the unknown causes of the reduced AFV in the study population. One possibility is an increase in fetal swallowing or decrease in fetal urinary output that is often observed in oligohydramniotic cases (Flack, Sepulveda, Bower, & Fisk, 1995). However, whether changes in fetal fluid balance other than the reduction in AFV occurred in this study is unclear so the origin of low AFV in these patients remains unresolved.

Our study provided new evidence for the biological functions and associated cellular pathways that presumably are altered in the amnion of oligohydramniotic patients. A consideration is that some of the pathways within individual categories of biological functions are activated while others are unchanged or suppressed under oligohydramnios conditions. The overall change in function would likely be a summation of the modifications in various pathways. A notable finding is the low fold change in differential expression of the protein mediators. This suggests that the modifications in pathway function may be subtle yet sufficient to produce the changes in AFV observed. Therefore, the causes of idiopathic oligohydramnios likely could be multifactorial and may include developmental and hereditary disorder, cellular or metabolic dysfunctions, and possibly consequences of comorbidities in pregnancy.

Oligohydramnios of unknown etiology often leads to adverse effects on maternal health and deleterious consequences in fetal survival. The present study implicated that idiopathic oligohydramnios is a complex obstetrical problem based on our present observations of functional modifications in several cellular pathways and mediators in the placental amnion under this condition. Disorders in cell and cytoskeletal assembly, immunological function and developmental disorder during pregnancy may be associated with low amniotic fluid. Furthermore, the up‐regulation of vesicular transport and intracellular trafficking mediators in the amnion of oligohydramniotic patients suggests that transport of amniotic fluid across the amnion cell layer would be enhanced thereby removing fluid from the amniotic compartment and resulting in reductions in AFV. Finally, the findings from the present study may provide the scientific basis for future pharmacologic interventions to normalize IM transport in amniotic fluid disorders.

## CONFLICT OF INTEREST

The authors declare that there are no conflicts of interest, and there are no disclosures to declare.

## References

[phy214381-bib-0001] Adams, E. A. , Choi, H. M. , Cheung, C. Y. , & Brace, R. A. (2005). Comparison of amniotic and intramembranous unidirectional permeabilities in late gestation sheep. American Journal of Obstetrics and Gynecology, 193, 247–255. 10.1016/j.ajog.2004.12.001 16021087

[phy214381-bib-0002] Anderson, D. F. , Jonker, S. S. , Louey, S. , Cheung, C. Y. , & Brace, R. A. (2013). Regulation of intramembranous absorption and amniotic fluid volume by constituents in fetal sheep urine. American Journal of Physiology: Regulatory, Integrative and Comparative Physiology, 305, R506–R511. 10.1152/ajpregu.00175.2013 PMC3763027 23824958

[phy214381-bib-0003] Benjamini, Y. , & Hochberg, Y. (1995). Controlling the false discovery rate: A practical and powerful approach to multiple testing. Journal of the Royal Statistical Society: Series B (Methodological), 57(1), 289–300. 10.1111/j.2517-6161.1995.tb02031.x

[phy214381-bib-0004] Brace, R. A. (1995). Progress toward understanding the regulation of amniotic fluid volume: Water and solute fluxes in and through the fetal membranes. Placenta, 16, 1–18. 7716121 10.1016/0143-4004(95)90077-2

[phy214381-bib-0005] Brace, R. A. , Anderson, D. F. , & Cheung, C. Y. (2014). Regulation of amniotic fluid volume: Mathematical model based on intramembranous transport mechanisms. American Journal of Physiology: Regulatory, Integrative and Comparative Physiology, 307, R1260–R1273. 10.1152/ajpregu.00283.2014 PMC4233290 25186112

[phy214381-bib-0006] Brace, R. A. , Anderson, D. F. , & Cheung, C. Y. (2018). Regulation of amniotic fluid volume: Insights derived from amniotic fluid volume function curves. American Journal of Physiology: Regulatory, Integrative and Comparative Physiology, 315, R777–R789. 10.1152/ajpregu.00175.2018 PMC6230884 30024777

[phy214381-bib-0007] Brace, R. A. , Cheung, C. Y. , & Anderson, D. F. (2014). Inhibitor of intramembranous absorption in ovine amniotic fluid. American Journal of Physiology: Regulatory, Integrative and Comparative Physiology, 306, R185–R189. 10.1152/ajpregu.00469.2013 PMC3921303 24381178

[phy214381-bib-0008] Cabrales Fontela, Y. , Kadavath, H. , Biernat, J. , Riedel, D. , Mandelkow, E. , & Zweckstetter, M. (2017). Multivalent cross‐linking of actin filaments and microtubules through the microtubule‐associated protein Tau. Nature Communications, 8, 1981. 10.1038/s41467-017-02230-8 PMC5719408 29215007

[phy214381-bib-0009] Cen, J. , Lv, L. , Wei, Y. , Deng, L. , Huang, L. E. , Deng, X. , … Pang, L. (2020). Comparative proteome analysis of amniotic fluids and placentas from patients with idiopathic polyhydramnios. Placenta, 89, 67–77. 10.1016/j.placenta.2019.10.010 31704631

[phy214381-bib-0010] Cheung, C. Y. , Anderson, D. F. , & Brace, R. A. (2016). Aquaporins in ovine amnion: Responses to altered amniotic fluid volumes and intramembranous absorption rates. Physiological Reports, 4(14), e12868 10.14814/phy2.12868 27440743 PMC4962073

[phy214381-bib-0011] Cheung, C. Y. , Anderson, D. F. , & Brace, R. A. (2017). Transport‐associated pathway responses in ovine fetal membranes to changes in amniotic fluid dynamics. Physiological Reports, 5(20), e13455 10.14814/phy2.13455 29051303 PMC5661228

[phy214381-bib-0012] Cheung, C. Y. , Anderson, D. F. , & Brace, R. A. (2019). Multiomics analyses of vesicular transport pathway‐specific transcripts and proteins in ovine amnion: Responses to altered intramembranous transport. Physiological Genomics, 51, 267–278. 10.1152/physiolgenomics.00003.2019 31150314 PMC6689731

[phy214381-bib-0013] Cheung, C. Y. , Beardall, M. K. , Anderson, D. F. , & Brace, R. A. (2014). Prostaglandin E_2_ regulation of amnion cell vascular endothelial growth factor expression: Relationship with intramembranous absorption rate in fetal sheep. American Journal of Physiology: Regulatory, Integrative and Comparative Physiology, 307, R354–R360. 10.1152/ajpregu.00070.2014 PMC4121629 24898841

[phy214381-bib-0014] Cheung, C. Y. , & Brace, R. A. (2008). Unidirectional transport across cultured ovine amniotic epithelial cell monolayer. Reproductive Sciences, 15, 1054–1058. 10.1177/1933719108322426 19088374

[phy214381-bib-0015] Flack, N. J. , Sepulveda, W. , Bower, S. , & Fisk, N. M. (1995). Acute maternal hydration in third‐trimester oligohydramnios: Effects on amniotic fluid volume, uteroplacental perfusion, and fetal blood flow and urine output. American Journal of Obstetrics and Gynecology, 173, 1186–1891. 10.1016/0002-9378(95)91350-5 7485317

[phy214381-bib-0016] Gilbert, W. M. , Eby‐Wilkens, E. , & Tarantal, A. F. (1997). The missing link in rhesus monkey amniotic fluid volume regulation: Intramembranous absorption. Obstetrics and Gynecology, 89, 462–465. 10.1097/00006250-199703000-00029 9052606

[phy214381-bib-0017] Hopkinson, A. , McIntosh, R. S. , Shanmuganathan, V. , Tighe, P. J. , & Dua, H. S. (2006). Proteomic analysis of amniotic membrane prepared for human transplantation: Characterization of proteins and clinical implications. Journal of Proteome Research, 5, 2226–2235. 16944934 10.1021/pr050425q

[phy214381-bib-0018] Locatelli, A. , Vergani, P. , Toso, L. , Verderio, M. , Pezzullo, J. C. , & Ghidini, A. (2004). Perinatal outcome associated with oligohydramnios in uncomplicated term pregnancies. Archives of Gynecology and Obstetrics, 269, 130–133. 10.1007/s00404-003-0525-6 12928935

[phy214381-bib-0019] Luo, B. H. , Carman, C. V. , & Springer, T. A. (2007). Structural basis of integrin regulation and signaling. Annual Review of Immunology, 25, 619–647. 10.1146/annurev.immunol.25.022106.141618 PMC1952532 17201681

[phy214381-bib-0020] Mann, S. E. , Nijland, M. J. , & Ross, M. G. (1996). Mathematic modeling of human amniotic fluid dynamics. American Journal of Obstetrics and Gynecology, 175, 937–944. 10.1016/S0002-9378(96)80028-7 8885751

[phy214381-bib-0021] Mills, I. G. , Praefcke, G. J. , Vallis, Y. , Peter, B. J. , Olesen, L. E. , Gallop, J. L. , … McMahon, H. T. (2003). Epsin R: An AP1/clathrin interacting protein involved in vesicle trafficking. Journal of Cell Biology, 160, 213–222. 12538641 10.1083/jcb.200208023 PMC2172650

[phy214381-bib-0022] Park, S. Y. , & Guo, X. (2014). Adaptor protein complexes and intracellular transport. Bioscience Reports, 34(4), e00123 10.1042/BSR20140069 24975939 PMC4114066

[phy214381-bib-0023] Park, S.‐J. , Yoon, W.‐G. , Song, J.‐S. , Jung, H. S. , Kim, C. J. , Oh, S. Y. , … Nirasawa, T. (2006). Proteome analysis of human amnion and amniotic fluid by two‐dimensional electrophoresis and matrix‐assisted laser desorption/ionization time‐of‐flight mass spectrometry. Proteomics, 6, 349–363. 10.1002/pmic.200500084 16294308

[phy214381-bib-0024] Phelan, J. P. , Smith, C. V. , Broussard, P. , & Small, M. (1987). Amniotic fluid volume assessment using the four‐quadrant technique in the pregnancy between 36–42 weeks. Journal of Reproductive Medicine, 32, 540–542. 3305930

[phy214381-bib-0025] Plubell, D. L. , Wilmarth, P. A. , Zhao, Y. , Fenton, A. M. , Minnier, J. , Reddy, A. P. , … Pamir, N. (2017). Extended multiplexing of tandem mass tags (TMT) labeling reveals age and high fat diet specific proteome changes in mouse epididymal adipose tissue. Molecular & Cellular Proteomics, 16, 873–890. 10.1074/mcp.M116.065524 28325852 PMC5417827

[phy214381-bib-0026] Sharshiner, R. , Brace, R. A. , & Cheung, C. Y. (2017). Vesicular uptake of macromolecules by human placental amniotic epithelial cells. Placenta, 57, 137–143. 10.1016/j.placenta.2017.06.344 28864003 PMC5657563

[phy214381-bib-0027] Shima, T. , Morikawa, M. , Kaneshiro, J. , Kambara, T. , Kamimura, S. , Yagi, T. , … Hirokawa, N. (2018). Kinesin‐binding‐triggered conformation switching of microtubules contributes to polarized transport. Journal of Cell Biology, 217, 4164–4183. 10.1083/jcb.201711178 30297389 PMC6279379

[phy214381-bib-0028] Spiro, J. E. , Konrad, M. , Rieger‐Fackeldey, E. , Masjosthusmann, K. , Amler, S. , Klockenbusch, W. , & Schmitz, R. (2015). Renal oligo‐ and anhydramnios: Cause, course and outcome–a single‐center study. Archives of Gynecology and Obstetrics, 292, 327–336. 10.1007/s00404-015-3648-7 25676656

[phy214381-bib-0029] Zhu, X. Q. , Jiang, S. S. , Zhu, X. J. , Zou, S. W. , Wang, Y. H. , & Hu, Y. C. (2009). Expression of aquaporin 1 and aquaporin 3 in fetal membranes and placenta in human term pregnancies with oligohydramnios. Placenta, 30, 670–676. 10.1016/j.placenta.2009.05.010 19545896

